# Irritable Bowel Syndrome Prevalence among Participants of Woodstock Rock Festival in Poland Based on Rome IV Criteria Questionnaire

**DOI:** 10.3390/ijerph182111464

**Published:** 2021-10-31

**Authors:** Joanna Palma, Justyna Antoniewicz, Krzysztof Borecki, Karol Tejchman, Karolina Skonieczna-Żydecka, Dominika Maciejewska-Markiewicz, Karina Ryterska, Natalia Komorniak, Maja Czerwińska-Rogowska, Anna Wolska, Honorata Mruk-Mazurkiewicz, Anna Gudan, Tomasz Mazur, Przemysław Mijal, Robert Budawski, Zofia Stachowska, Wojciech Marlicz, Ewa Stachowska

**Affiliations:** 1 Department of Biochemical Sciences, Pomeranian Medical University in Szczecin, 71-460 Szczecin, Poland; joanna.palma@pum.edu.pl (J.P.); k.borecki.pum@gmail.com (K.B.); karzyd@pum.edu.pl (K.S.-Ż.); honoratamruk15@gmail.com (H.M.-M.); 2 Department of Human Nutrition and Metabolomics, Pomeranian Medical University in Szczecin, 71-460 Szczecin, Poland; justynakaldunska@wp.pl (J.A.); dmaciejewska.pum@gmail.com (D.M.-M.); karina.ryterska@pum.edu.pl (K.R.); natalia.komorniak@pum.edu.pl (N.K.); majaczerwinska89@gmail.com (M.C.-R.); wolskaanna92@gmail.com (A.W.); gudananna@gmail.com (A.G.); tomasz.mazur90@gmail.com (T.M.); przemyslaw.mijal@gmail.com (P.M.); rbudawski93@gmail.com (R.B.); ewast@pum.edu.pl (Z.S.); 3 Department of General Surgery and Transplantation, Pomeranian Medical University in Szczecin, 70-204 Szczecin, Poland; karol.tejchman@pum.edu.pl; 4 Department of Gastroenterology, Pomeranian Medical University in Szczecin, 71-252 Szczecin, Poland; marlicz@hotmail.com

**Keywords:** functional gastrointestinal disorder (FGID), gastrointestinal tract

## Abstract

Background: Irritable bowel syndrome (IBS) is a chronic functional gastrointestinal disorder (FGID), in which etiology and pathogenesis are not fully explored. There is an ongoing need for more population studies adhering to new ROME IV criteria. In the current study, which follows our previous investigation among participants of the Woodstock Rock Festival in Poland, we aimed to evaluate the prevalence of IBS and its relation to age, gender, education, and IBS type. Methods: Rome IV criteria questionnaire was used to assess abdominal complaints of 386 participants of the Woodstock Rock festival 2018. Results: Analyzed data revealed that Rome IV criteria were met by 42 participants (11.41%), 11 men and 31 women (*p* = 0.0028), with following types of IBS: IBS-M (mixed form) 55%, IBS-D (with diarrhea) 33%, IBS-U (unclassified) 10%, IBS-C (with constipation) 2%. No statistically significant correlation between IBS prevalence and age, gender, or education (*p* > 0.05) was found. Conclusions: The prevalence of IBS among major rock festival participants in Poland was high. Women met the criteria more often than men, which is consistent with global epidemiology for many years. Among participants of the Woodstock Rock Festival, the most frequent subtype was IBS-M, the rarest—IBS-C. There is a need of conducting cohort studies in bigger groups in our population.

## 1. Introduction

Functional gastrointestinal disorders (FGIDs) or disorders of gut-brain interaction (DGBIs) [1,2]—including functional dyspepsia (FD) and irritable bowel syndrome (IBS) are increasingly diagnosed worldwide [3]. The diagnosis of IBS is based mainly on the patient’s history and clinical symptoms [4,5] and is characterized by recurrent abdominal pain, changed pattern and frequency of defecation, flatulence, and lack of alarm symptoms [6,7]. In some patients, those symptoms are mild or moderate, in others quite intensive, with a negative impact on the quality of life (QoL). IBS prevalence depends on many factors, including: (i) past infectious diseases of the gastrointestinal tract, (ii) clinical or laboratory confirmation of bowel inflammation, diagnostics criteria used, and geographic location (race, culture, economics) [8]. In many studies, authors stress the importance of age [9,10] and gender [11] on the frequency of IBS occurrence. IBS etiology is still not well understood, however strictly connected with altered: (i) intestinal motility, (ii) visceral sensitivity, (iii) mucosal immune function, and (iv) microbiota, which may result from numerous environmental insults (e.g., diet, stress, air pollution, infection).

New studies along with clinical, molecular, or metagenomics assessments, include also the evaluation of psychosocial symptom [12], which reflects the functioning of the gut-brain axis assessed by new Rome IV criteria, published in 2016 [1,2] The most recent criteria stands out four main subtypes of IBS: with constipation (IBS-C), with diarrhea (IBS-D), mixed (IBS-M) and unclassified (IBS-U), worked out on stool consistency evaluated according to Bristol Stool Form Scale [13]. Upgrades included in Rome IV criteria regard mainly the terminology of IBS diagnostics criteria [14]. It is stressed however that the value of those criteria is connected with the evaluation of IBS prevalence in a certain population and by that, the unification of collected data methodology. It makes it possible to understand the problems of global IBS epidemiology as well as to compare statistics [15].

In the current article, we focused our attention on IBS prevalence among participants of the Woodstock Rock Festival in Poland. Our previous research [10,16] revealed that in the examined group people with chronic gastrointestinal disorders can be identified. In the current study, we focused on analyzing IBS prevalence in relation to age, gender, and education using a questionnaire based on the Rome IV criteria.

## 2. Materials and Methods

### 2.1. Examined Group

The research was conducted on the population of Woodstock Rock festival participants in the year 2018 in Poland. The examined group consisted of 386 people of the Caucasian race from Poland, 178 (48.37%) men and 190 women (51.63%). The age range was 18–62, mean 27.10 ± 8.67 years, median 24.00 years (IQR 21.00–30.00). Data were collected on mobile tablet/phone applications. Questionnaires were anonymous. Written consent was not required which was confirmed by the Bioethics Committee of the Pomeranian Medical University. At the moment of the recruitment, participants gave only verbal consent for the questionnaire.

### 2.2. Questionnaire

The questionnaire was designed based on Rome IV criteria, with a focus to evaluate the prevalence of IBS and its subtypes and to point its relation to age, gender and education. The questionnaire also included demographic questions (age, gender, educational stage).

According to the Rome IV criteria, the irritable bowel syndrome (IBS) is identified based on diagnostic criteria (https://theromefoundation.org/ accessed on 11 July 2017) [17] which include abdominal pain on average at least one day in a week in the last 3 months, connected to two or more of the following aspects:

1. Related to defecation
2. The change in the frequency of stool
3. The change in the form (appearance) of stool

The Bristol Stool Form Scale was used to evaluate IBS subtypes.

### 2.3. Statistical Analysis

Quantitative data distribution were verified by Shapiro-Wilk Test for Normality in R (R Foundation for Statistical Computing, Vienna, Austria). Qualitative data were analyzed with two way Fisher’s exact test. Quantitative data were analyzed with Mann–Whitney test. All analyses were performed using GraphPad Prism v.8.02 (GraphPad Software Inc., San Diego, CA, USA). The statistical level of significance was set at *p* < 0.05.

## 3. Results

In the examined group (*n* = 386), 48.37% of participants declared higher education, 48.10% medium education, 2.17% basic education, 1.36% vocational education. The gross enrolment ratio for higher education institutions in 2019/20 was 46.6% in the Polish population [18]. IBS Rome IV criteria were met by 42 participants, 11 men (48.77%) and 31 women (73.81%), difference in percentage was statistically significant (*p* = 0.0028; OR (95% CI) = 2.96 (1.44–6.16)). Median age was lower in IBS group (23.50) than in group without IBS (25.00), however statistical significance was not met (*p* > 0.05). Participants with IBS (*n* = 42) declared higher (42.86%) or medium education (57.14%). Participants without IBS (*n* = 313) declared higher (49.08%), medium (46.93%), basic (2.45%) and vocational education (1.54%). Observed differences in education was statistically insignificant (*p* > 0.05).

Demographic data and the analysis of age, gender, and education on IBS prevalence were presented in Table 1.

Using Bristol Stool Form Scale in the questionnaire, we recorded the following types of IBS: IBS-C 2.38%, IBS-D 33.33%, IBS-M 54.76%, and IBS-U 9.53%. It should be mentioned that only among women IBS-U (*n* = 4) and IBS-C (*n* = 1) were labeled. Moreover, women more frequently than men were classified to the IBS-D group (57.14% vs. 42.86%) and (57.14% vs. 42.86%). Median participant age with IBS-C or IBS-D (*n* = 15) was 23 years (IQR 21.00–25.00). Median participant age with IBS-M or IBS-U (*n* = 27) was 24 years (IQR 22.00–32.00). Medium education was the most frequently declared either in the group with IBS-C or IBS-D (60.00%) and in the group with IBS-M or IBS-U (55.56%). Participants without symptoms of IBS did not declare basic or vocational education. Analysis revealed no statistical significance between the IBS subtype and age, gender as well as the participants’ education (*p* > 0.05).

Demographic data with main IBS subtypes and statistical analysis of its relation to age, gender, and education were presented in Table 2.

## 4. Discussion

Our study is a continuation of our previous research. Currently, we aimed to evaluate the prevalence of IBS among participants of the 2018 Woodstock Rock festival in Poland, using the new questionnaire based on Rome IV criteria. We identified IBS symptoms, fulfilling Rome IV criteria in 11% (*n* = 42) of all respondents. Our results, supplement the latest data [3] on the IBS prevalence in the Polish population. IBS-M was declared by 55% respondents (*n* = 23), IBS-D 33% (*n* = 14), IBS-U 10% (*n* = 4), and IBS-C 2% (*n* = 1). Moreover, we aimed to verify the relation between IBS subtypes and age, gender as well as education. Collected data indicates that IBS symptoms were significantly more frequent in women (*p* = 0.0028; OR (95% CI = 2.96 (1.44–6.16)). There was no connection between IBS and age or the level of education. There was also no association between gender, age, or the level of education and IBS subtypes (*p* > 0.05).

One of the first studies which evaluated IBS prevalence was performed by Hungin et al. [19] on almost 42,000 participants from 8 European countries. Using a telephone poll based on a validated questionnaire accordant to Rome I, II, and Manning criteria showed that IBS prevalence equals 11.5%. Since that study many others appeared, where IBS prevalence was determined in certain countries [20,21,22,23,24,25,26]. Studies using Rome I, II, and Manning criteria determined, that global IBS prevalence equals 10–15% [15,27], 5–10% was addressed in most European countries, the USA, and China [6]. Rome IV criteria implementation decreased by half the prevalence of IBS [28,29]. The newest Sperber et al. [3] study revealed that IBS prevalence reaches 3–5% in 19 of 26 examined countries (Poland at 4.4%). This result is significantly lower than in our study, which might be caused by the method of the questionnaire, internet surveys, and the group size. The question remains if those differences are caused by methodology or by the actual prevalence of IBS in the population [3,15,30]. According to Rome IV criteria, abdominal pain should occur at least once a week during the last 3 months [31]. Moreover, the term "discomfort" was eliminated in the new IBS definition, as unspecific, unprecise, and with different meanings in various languages [14]. Next, the statement that pain is related to changed stool pattern and its consistency was also omitted. Earlier criteria encompassed more broad definitions related to defecation pain patterns [31]. Therefore upgrade in Rome criteria (IV) may influence the overall prevalence of IBS. However as investigated by Sperber et al. [3] in their most recent large-scale multi-national study, run across 33 countries, more than 40% of persons worldwide suffer from various FGIDs/DGBIs. Likely, the time devoted to answering all questions in a very extensive and long ROME IV questionnaire impacts the quality of responses.

In one of the earliest studies on IBS, it was shown, that subtypes IBS-C and IBS-D are the most common [19]. IBS-C predominates in women and IBS-D in men [32]. Our results indicate that among examined Polish participants, the predominant type is IBS-M and IBS-D. Both types are more frequent among women than men: IBS-D (57.14% vs. 42.86%), IBS-M (78.26% vs. 21.74%). Additionally, among women, we showed the prevalence of IBS-U (*n* = 4) and IBS-C (*n* = 1). It differs from results achieved by Sperber et al. [3], where authors observed that IBS-C on a global scale is predominant among women and IBS-D among men, which is consistent with studies in the general population. It must be emphasized that gathering data about certain types of IBS is complex. Apart from IBS evaluation methods, data firmly show, that disease and FGIDs symptoms are more frequent among women and they can be present during a puberty period, which may be influenced by hormones regulating physical stress and bowel peristalsis [3,8,27,32,33,34].

Present data also show that IBS prevalence lowers with the age [6]. Our previous studies based on II and III Rome criteria revealed, that IBS prevalence was predominant among young women [10]. The implementation of Rome IV criteria, however, did not allow us to confirm the statistical significance of the above correlations. It seems that methods of our analysis and size of the studied group may be critical for IBS diagnosis, while it is stressed that the new criteria restrict the number of qualified cases [29]. Right now, this is one of the broadest studies of the prevalence of IBS in the Polish population based on the newest criteria. So far, during the research conducted among the participants of the festival, we have observed that young adult inhabitants of Poland often struggle with FGIDs [10]. Other studies we conducted indicated that gut-brain disruptions have a significant influence on these symptoms [16]. In this case, we paid particular attention to the fact that insomnia and depressive behavior are prevalent in the Polish population [35].

IBS prevalence varies between certain countries and in its estimation depends on many factors, mainly examination method, group size, group selection, and type of questionnaire [3,19,30]. The results of numerous studies indicate that educational level and marital status also influence the presence of IBS [36,37,38]. To a large extent, they reflect the self-awareness of one’s health and social-psychological status. Particularly in women, social status (being single), lower education, lower income, or being unemployed translate into a higher prevalence of IBS [37]. Moreover, the development status of countries also has a large impact on IBS prevalence and diagnosis [15]. Data regarding IBS prevalence in African and Asian countries are rare and hard to interpret [39]. There is a continuous need to collect the data on a global scale, which could help to better understand the epidemiology and etiology of FGIDs/DGBIs. It is stressed that, until new criteria are released, the data should be collected according to Rome IV criteria, to unify the methodology of newly designed studies and to perform adequate comparisons. New criteria could substantially improve future research and therefore develop health service and their medical resources [15,30]. It is important, especially in the era of Covid-19 and future pandemics, which can significantly change the prevalence of FGIDs/DGBIs. The studies investigating the impact of the current pandemic on mental and physical status are ongoing [40].

Our study has some limitations. The applied non-probability sample method and the small number of participants in the group could have an impact on the result of the study with reference to the population of the country. The research was conducted during the Woodstock Rock festival in 2018 in Poland, the attendees of which might be considered as a specific group of persons. However, the participants originated from all regions of Poland, hence they could to at least some extent, constitute a representative sample for the country. The study included randomly selected participants of the festival and each eligible study responder volunteered to participate in the experiment.

## 5. Conclusions

The prevalence of IBS as assessed with the ROME IV questionnaire among major Woodstock Rock 2018 festival in Poland was 11.42%. Women more frequently suffer from IBS symptoms, which is in line with our previous results based on Rome II and III criteria. The prevalence of IBS in the studied population in Poland was higher than other European countries recently reported. Age and level of education had no statistically significant influence on the frequency of IBS. There is a need for a population screening according to Rome IV criteria to unify existing data and to perform adequate comparisons.

## Figures and Tables

**Table 1:** Demographic data and the analysis of age, gender, and education on IBS prevalence.

Category Title	All, *n* = 368		IBS, *n* = 42 (11.41%)		Non-IBS, *n* = 326 (88.59%)		IBS vs. Non-IBS		
							*p*	OR (95%CI)	
Gender									
Male, *n* (%)	178	(48.37)	11	(26.19)	167	(51.23)	—	1.00	
Female, *n* (%)	190	(51.63)	31	(73.81)	159	(48.77)	**0.0028**	2.96	(1.44–6.16)
Age (year)									
Min-Max	18–62		18–50		18–62		—	—	
Mean (± SD)	27.10	(8.67)	25.93	(7.72)	27.25	(8.79)	—	—	
Median (IQR)	24.00	(21.00–30.00)	23.50	(22.00–26.25)	25.00	(21.00–30.00)	0.4248 *	—	
Education									
Higher or Secondary, *n* (%)	355	(96.47)	42	(100.00)	313	(96.01)	0.3769	Infinity	(0.43-Infinity)
Non-Higher, Non-Secondary, *n* (%)	13	(3.53)	0	(0.00)	13	(3.99)	—	1.00	
Higher, *n* (%)	178	(48.37)	18	(42.86)	160	(49.08)	0.5130	0.78	(0.41–1.49)
Non-Higher, *n* (%)	190	(51.63)	24	(57.14)	166	(50.92)	—	1.00	
Secondary, *n* (%)	177	(48.10)	24	(57.14)	153	(46.93)	0.2515	1.51	(0.79–2.86)
Non-Secondary, *n* (%)	191	(51.90)	18	(42.86)	173	(53.07)	—	1.00	
Vocational, *n* (%)	5	(1.36)	0	(0.00)	5	(1.53)	>0.9999	0.00	(0.00–5.34)
Non-Vocational, *n* (%)	363	(98.64)	42	(100.00)	321	(98.47)	—	1.00	
Primary, *n* (%)	8	(2.17)	0	(0.00)	8	(2.45)	0.6045	0.00	(0.00–3.59)
Non-Primary, *n* (%)	360	(97.83)	42	(100.00)	318	(97.55)	—	1.00	

95% CI—95% confidence interval; IBS—Irritable bowel syndrome; IQR—interquartile range; OR—odds ratio; *p*—*p* value in two-sided Fisher’s exact test or two-sided Mann–Whitney U test (marked with *); SD—standard deviation; statistically significant *p* values (*p* < 0.05) are bolded. IBS with constipation (IBS-C), IBS with diarrhea (IBS-D), mixed IBS (IBS-M), and unclassified IBS (IBS-U).

**Table 2:** Demographic data with main IBS subtypes symptoms declaration and statistical analysis of its relation to the age, gender, and education.

IBS Total *n* = 42	IBS-C, *n* = 1 (2.38%)		IBS-D, *n* = 14 (33.33)		IBS-M, *n* = 23 (54.76)		IBS-U, *n* = 4 (9.53)		IBS-D vs. IBS-M			IBS-C/D vs. IBS-M/U		
									*p*	OR (95%CI)		*p*	OR (95%CI)	
Gender														
Males, *n* (%)	0	(0.00)	6	(42.86)	5	(21.74)	0	(0.00)	—	1.00		—	1.00	
Females, *n* (%)	1	(100.00)	8	(57.14)	18	(78.26)	4	(100.00)	0.2679	0.37	(0.08–1.45)	0.1582	0.34	(0.08–1.26)
Age (year)														
Min-Max	—		18–27		19–50		22–27		—	—		—	—	
Mean (± SD)	21	—	23.07	(2.53)	28.13	(9.72)	24.50	(2.89)	—	—		—	—	
Median (IQR)	21	—	23.00	(21.00–25.00)	24.00	(22.00–32.00)	24.50	(22.00–27.00)	0.2361 *	—		0.1169 *	—	
Education														
Higher, *n* (%)	0	(0.00)	6	(42.86)	10	(43.48)	2	(50.00)	>0.9999	0.98	(0.29–4.03)	>0.9999	0.83	(0.25–2.94)
Secondary, *n* (%)	1	(100.00)	8	(57.14)	13	(56.52)	2	(50.00)	—	1.00		—	1.00	
Vocational, *n* (%)	0	(0.00)	0	(0.00)	0	(0.00)	0	(0.00)	—	—		—	—	
Primary, *n* (%)	0	(0.00)	0	(0.00)	0	(0.00)	0	(0.00)	—	—		—	—	

95%CI—95% confidence interval; IBS—Irritable bowel syndrome; IBS-C—constipation-predominant IBS; IBS-D—diarrhea-predominant IBS; IBS-M—mixed IBS; IBS-U—unclassified IBS; IQR—interquartile range; OR—odds ratio; *p*—*p* value in two-sided Fisher’s exact test or two-sided Mann—Whitney U test (marked with *); SD—standard deviation.
